# Survival of L. casei DG^®^ (*Lactobacillus paracasei* CNCMI1572) in the gastrointestinal tract of a healthy paediatric population

**DOI:** 10.1007/s00394-018-1860-5

**Published:** 2018-11-29

**Authors:** Milko Radicioni, Ranjan Koirala, Walter Fiore, Chiara Leuratti, Simone Guglielmetti, Stefania Arioli

**Affiliations:** 1 CROSS Research S.A., via F.A. Giorgioli 14, 6864 Arzo, Switzerland; 2 SOFAR SpA, 20060 Milan, Trezzano Rosa Italy; 3 Department of Food, Environmental and Nutritional Science (DeFENS), University of Milan, Milan, Italy

**Keywords:** Healthy children, Probiotics, L. casei DG^®^ recovery, *Lactobacillus paracasei* CNCMI1572

## Abstract

**Purpose:**

Ability to survive the digestive process is a major factor in determining the effectiveness of a probiotic. In this study, the ability of the probiotic L. casei DG^®^ (*Lactobacillus paracasei* CNCMI1572) to survive gastrointestinal transit in healthy children was investigated for the first time.

**Methods:**

Twenty children aged 3–12 years received L. casei DG^®^ as drinkable solution of 1 × 10^9^ colony forming units (CFU), once daily for 7 consecutive days. Recovery in faecal samples was evaluated at baseline and at different time-points during and after administration. Defecation frequency, faeces consistency, digestive function and product safety were also assessed.

**Results:**

Nineteen (95%) of the 20 enrolled children presented viable L. casei DG^®^ cells in their faeces at least once during the study, with a maximum count (mean 4.3 log_10_ CFU/g ± 2.3) reached between day 4 and 6 from the beginning of consumption. Notably, for 11 (57.9%) of the 19 children with viable cells, L. casei DG^®^ survived in faecal samples up to 3 days after treatment end. Defecation frequency, faeces consistency and digestive function did not change considerably during or after study treatment. Safety of the study product was very good.

**Conclusions:**

This study showed for the first time that L. casei DG^®^ survives the gastrointestinal transit when ingested by children with a paediatric probiotic drinkable solution containing 1 × 10^9^ CFU, and persists in the gut up to 3 days after the end of product intake, demonstrating resistance to gastric juices, hydrolytic enzymes and bile acids.

**Electronic supplementary material:**

The online version of this article (10.1007/s00394-018-1860-5) contains supplementary material, which is available to authorized users.

## Introduction

A first assessment of probiotics efficacy was made in 2001 by an International Expert Consultation group, working for the Food and Agricultural Organization (FAO) of the United Nations and the World Health Organization (WHO), resulting in the Guideline for the Evaluation of Probiotics in Food, published in 2002 [1]. One output was a reworking of the definition of probiotics, which was accepted in 2014 by the International Scientific Association for Probiotics and Prebiotics [2], with only a minimal grammatical change, as follows: “Probiotics are live microorganisms that, when administered in adequate amounts, confer a health benefit on the host”.

The health promoting effects of probiotic bacteria, mostly lactobacilli and bifidobacteria, are being increasingly reported, in particular in patients affected by pathological conditions [1–7]. In a very recent review on the role of probiotics, Khalesi et al. [8] confirmed that probiotic supplementation generates a transient improvement in gut microbiota and has a role in improving immune system responses, stool consistency, bowel movement and vaginal lactobacilli concentration also in healthy subjects. In addition, the authors confirmed that in healthy adults probiotic consumption can have a beneficial effect on the immune, gastrointestinal and female reproductive health systems.

An effective probiotic should be preferably of human origin, remain viable during storage and use, be generally recognized as safe (GRAS), confer health benefits on the host, modulate host immunity, prevent or treat a specific pathogen infection by antimicrobial production, adhere to human intestinal cells, contain a large number of viable cells and be capable of surviving in the gut [5]. It follows that a major factor in determining the effectiveness of a probiotic is its ability to survive the digestive process and thrive in the gastrointestinal tract [9–13]. In the gut, in fact, ingested bacteria are confronted with many physicochemical effects that may adversely influence bacteria viability. These include gastric acid, bile acid and digestive enzymes, along with the highly diverse and competitive environment presented by the gut microflora [14, 15].

Interestingly, survival of different *lactobacilli* strains in the gastrointestinal tract after oral ingestion has been demonstrated in several faecal recovery studies conducted in healthy volunteers [16–18].

*Lactobacillus paracasei* is a normal component of healthy individuals’ intestinal microflora, commonly used in probiotics products. L. casei DG^®^ (*Lactobacillus paracasei* CNCMI1572; LCDG) is a probiotic strain isolated from human faeces and developed by SOFAR S.p.A. in the Enterolactis^®^ line products. LCDG was deposited at the Pasteur Institute, Paris (deposit N. CNCMI1572).

Characteristics of LCDG are its ability to adhere to the small intestine mucosae, to produce lactic acid, to survive under pH 3.0 conditions and in the presence of bile acids, and not to induce antibiotics resistance [19, 23].

Consistently with these peculiarities a number of in vitro/in vivo studies support its therapeutic use: in healthy adults LCDG was shown to have the ability to modulate the intestinal microbial ecosystem [20] and to influence host’s immune responses [21, 22] through its unique exopolysaccharide capsule [23]. In addition, LCDG is endowed with therapeutic potential for several dysfunctional and pathological conditions such as ulcerative colitis [24], diverticular disease [25, 26], small intestinal bacterial overgrowth [27] and irritable bowel syndrome [23, 28].

A previous study in healthy adult volunteers, administered an adult LCDG formulation containing 8.5 × 10^9^ CFU, once a day for 7 days, demonstrated the presence of live LCDG cells in the collected faeces up to 7 days after the end of treatment [29]. In the study by Ferrario et al. [20], LCDG cells in faecal samples of healthy adults were significantly increased as compared to baseline after 4-week once daily administration of capsules (Enterolactis^®^ Plus) containing at least 24 × 10^9^ viable cells. The same study also demonstrated that the intake of LCDG modulated gut microbiota, in particular by increasing the Costridiales geni *Coprococcus:Blautia* ratio, which, according to the literature, could potentially confer a health benefit on the host. More recently, LCDG was found to be able to survive after passage through the gastrointestinal tract in healthy adults [30].

The aim of the present open-label, 1-week treatment study was to confirm the ability of an LCDG paediatric formulation, containing 1 × 10^9^ live bacteria, to transit alive through the gastrointestinal tract in children during and after the administration period. Product safety, defecation frequency, faeces consistency and digestive function were also evaluated.

## Methods

### Study design and participants

**Fig. 1:**
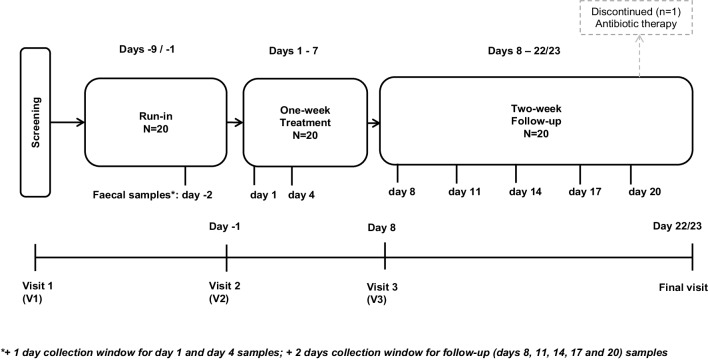
Graphic representation of the study design

This was a single centre, open-label, one-arm, recovery study, which included a screening visit, a 1-week run-in, a 1-week administration period, a 2-week follow-up period and a final visit. After the screening visit (V1), subjects attended the clinical centre on the day before the first administration (day − 1, V2), on day 8 (V3) and for the final visit (day 22/23) (Fig. 1).

The study protocol (no. PSC-DS RECENT-BS 16) was approved by the Ethics Committee of Canton Ticino, Switzerland. All the subjects were given a detailed description of the study and all of them gave written informed consent before enrolment. The study was performed from August to October 2017, in accordance with the Declaration of Helsinki, harmonised European standards for Good Clinical Practice (ICH E6 1.24) and the applicable local laws.

Healthy male and female children, aged 3–12 years and classified as not overweight based on the body mass index chart for sex and age [31], were enrolled in the study. All children were in good physical health, as assessed through a full physical examination at screening. No subjects were on abnormal diets or vegetarians. Children with a defecation frequency above 3 stools per day or less than 3 stools per week were not enrolled. Exclusion criteria also included the following: history or presence of significant diseases, in particular inflammatory/infective intestinal diseases, viral or bacterial enteritis, gastric or duodenal ulcer, metabolic diseases, primary or secondary immunodeficiency; antibiotics intake within 1 month before the screening visit; any other medication, including over the counter drugs, for 2 weeks before the study. Subjects were not enrolled if they were hypersensitive or allergic to any study product’s ingredient or food components and if they had participated in other clinical trials in the past 3 months.

### Investigational product

Enterolactis^®^ is a probiotic formulation based on L. casei DG^®^ (= *Lactobacillus paracasei* CNCMI1572 = LCDG viable cells). The product was supplied as vials containing 1 × 10^9^ CFU as powder in the cap (SOFAR SpA, Italy) and a 2% fructose solution (additives: citric acid as acidity controller, and sodium benzoate and potassium benzoate as preservatives).

All children enrolled in the study received one vial of the investigational product, once daily from day 1 to day 7.

The product was reconstituted just before intake. Upon opening of the vial, the powder in the cap directly mixed with the drinkable solution. For the intake, after the vial was shaken, the children drank the content of the vial directly, under fasting conditions, in the morning at least 10 min before breakfast, or alternatively in the evening before going to bed, at least 2 h after the last meal of the day. Administrations date and time were recorded on a daily diary. Product accountability and diary check were performed to check treatment compliance.

During the entire study, the subjects continued their normal diet except for fermented milk, probiotics food supplements or any other probiotic-containing products and prebiotics food supplements, which were forbidden from the start of the run-in phase until study end. Traditional yoghurts were allowed. The intake of any medication was reported as a protocol deviation.

### Faecal sample collection and analysis

Faecal samples were collected at baseline (day − 2), during the 1-week treatment (day 1 and 4) and at follow-up (day 8, 11, 14, 17 and 20). Collection times could vary of + 1 day at baseline or + 2 days at all the other time-points. Samples were collected in sterile containers, stored at home at approximately 2–8 °C, picked up by a courier as soon as possible after defecation and delivered at 2–8 °C to the Department of Food, Environmental and Nutritional Sciences (DeFENS), University of Milan, Italy.

Each fresh faecal sample was processed immediately after the delivery to the laboratory, that is within 24 h after defecation, in order not to affect the viability of the probiotic strain. The protocol for the analysis is described in Arioli et al. [30]. Specifically, after homogenization of the sample, 1 g of faeces was resuspended in 9 mL Maximum Recovery Diluent (MRS; Scharlau) and mixed with a Stomacher. Then, the faecal suspension was serially 1:10 diluted and inoculated by spreading on agar plates containing MRS medium (Difco) supplemented with 1 mg/L vancomycin and 10 mg/L kanamycin (vkMRS). Finally, plates were incubated anaerobically at 37 °C for up to 48 h. The identification of the colonies as LCDG strain was carried out by assessing the sticky/filamentous texture of the colony and through an end point-colony PCR with strain specific primers (rtWELFf and rtWELFr) [20]. PCRs were performed in 25-µL reaction mixtures, each containing 1 colony (picked with a sterile wooden stick), 2.5 µL of 10 × reaction buffer, 200 µmol/L of each dNTP, 0.5 mmol/L MgCl_2_, 0.5 µmol/L each primer, and 0.5 U DreamTaqTM DNA polymerase (Thermo Fisher Scientific Inc., Monza, Italy). Amplifications were carried out using a Mastercycler 96 (Eppendorf, Milan, Italy). The PCR mixtures were subjected to the following thermal cycling conditions: initial hold at 95 °C for 3 min followed by 39 cycles of 95 °C for 30 s, 58 °C for 30 s and 72 °C for 30 s. Amplification products were resolved by electrophoresis on a 2% (w/v) agarose gel (with 0.2 µg/mL ethidium bromide) in 1 × TAE buffer (40 mmol/L Tris-acetate, 1 mmol/L EDTA, pH 8.0) and photographed. A 1-kb GeneRuler DNA Ladder Mix was used as a size marker. The method has a detection limit of 100 LCDG cells/g of wet faeces. Result values are presented as log_10_ CFU/g of wet faeces.

### Defecation frequency, stool consistency, digestive function and safety assessments

Besides investigational product administration date/time, study subjects or their parent(s) reported in a daily diary: defecation date/time, stool consistency, adverse events occurrence and concomitant medication intake. Stool consistency was assessed according to the illustrations associated with the 1–7 score system of the Bristol stool scale [32]. Scores were as follows: (1) separate hard lumps like nuts; (2) sausage-shaped but lumpy, (3) like a sausage but with cracks on the surface, (4) like a sausage or snake, smooth and soft; (5) soft blobs with clear-cut edges; (6) fluffy pieces with ragged edges, a mushy stool; (7) watery, no solid pieces, entirely liquid.

In addition, digestive function was evaluated daily in the diary as bad (score 1), normal (score 2), good (score 3) or optimal (score 4) from the day before first administration until day 8. Product intake global evaluation was assessed by the investigator on day 8.

Safety and general tolerability of the investigational product were based on treatment-emergent adverse events occurrence, daily diary check and physical examinations performed at screening and final visit.

### Sample size and data analysis

Study sample size was not based on any formal calculation but was deemed appropriate for the descriptive and pilot nature of the study.

The data documented in this trial and the parameters measured were described using classic statistics, i.e. mean, SD, CV (%), minimum and maximum values, for quantitative variables and frequencies for qualitative variables. Data not available were evaluated as “missing values”. The analysis was performed using SAS^®^ version 9.3 (TS1M1).

Adverse events were coded using the Medical Dictionary for Regulatory Activities version 20.1.

## Results

### Demography and disposition of the study participants

Twenty (20) healthy children, 10 males and 10 females, satisfying the study inclusion/exclusion criteria, were enrolled, received all planned doses of the investigational product and were included in the data analyses. Demographic characteristics of the study subjects are presented in Table 1.

**Table 1:** Demography of the study children

Parameter	Analysed subjects *N* = 20
Sex	
Male—*n* (%)	10 (50%)
Female—*n* (%)	10 (50%)
Race	
White	20 (100.0%)
Age (years)	
Mean ± SD	7.0 ± 2.8
Median (range)	6.5 (3–12)
Body weight (kg)	
Mean ± SD	27.07 ± 11.64
(Range)	25.05 (13.4–59.5)
Height (cm)	
Mean ± SD	125.1 ± 19.0
(Range)	125.0 (94–170)
Body mass index (kg/m^2^)	
Mean ± SD	16.49 ± 1.89
(Range)	15.75 (14.2–20.9)

Nineteen (19) children completed the study per protocol, while one (subject 12) discontinued the study during the follow-up phase, after completing the 1-week treatment period, due to an antibiotic therapy to cure a tooth abscess (i.e. azithromycin 180 mg suspension) not allowed according to the study requirements.

### L. casei DG^®^ (LCDG) faecal recovery

At baseline, no viable LCDG cells were present in the analysed faecal samples. This was expected considering that the children were instructed not to consume any probiotic/prebiotic food components or supplements.

During the administration period most subjects showed variable counts of live LCDG CFU in their faeces. In particular, viable cells of LCDG were isolated from at least one faecal sample in 19 (95%) of the 20 treated children, with the only exception of one child for whom no viable cells were detected (Tables 2, 3). Individual responses and demographic data are listed in Supplementary Table S1.

**Table 2:** Percentage of children with viable L. casei DG^®^ cells in faecal samples collected at baseline (day—2 [+ 1]), during treatment (Day 1 [+ 2]), Day 4 [+ 2]) and at follow-up (Day 8 [+ 2] and days 11, 14, 17 and 20 [+ 2])

Assessments	Subjects number	Subjects, *n* (%) with viable L. casei DG^®^ in faecal sample				
		Baseline	One-week treatment		Follow-up	
		Day − 2 (+ 1)	Day 1 (+ 2)	Day 4 (+ 2)	Day 8 (+ 2)	Day 11, 14, 17, 20 (+ 2)
Daily assessment	20	0 (0.0%)	3 (15.0%)	16 (80.0%)	11 (55.0%)	0 (0.0%)
Overall	20	0 (0.0%)	19 (95.0%)			0 (0.0%)

**Table 3:** Individual and mean (± SD) counts of viable L. casei DG^®^ in faecal samples of the study children (*N* = 20) at baseline, during the probiotic administration period and at follow-up

Subject	Viable L. casei DG^®^ counts (log10 CFU/g faeces)				
	Baseline	One-week administration period		Follow-up	
	Day − 2 (+ 1)	Day 1 (+ 2)	Day 4 (+ 2)	Day 8 (+ 2)	Days 11 (+ 2), 14 (+ 2), 17 (+ 2), 20 (+ 2)
1	BDL	BDL	5.7	3.7	BDL
2	BDL	BDL	4.5	BDL	BDL
3	BDL	BDL	BDL	5.5	BDL
4	BDL	BDL	5.7	BDL	BDL
5	BDL	BDL	BDL	4.7	BDL
6	BDL	BDL	4.7	BDL	BDL
7	BDL	BDL	5.9	BDL	BDL
8	BDL	BDL	5.3	4.7	BDL
9	BDL	4	6.3	4	BDL
10	BDL	BDL	3.7	4.7	BDL
11	BDL	BDL	5	3.95	BDL
12^a^	BDL	BDL	5.3	BDL	BDL^b^
13	BDL	BDL	5.9	3.3	BDL
14	BDL	BDL	5	4.7	BDL
15	BDL	4.8	5.3	4	BDL
16	BDL	BDL	5.9	4.3	BDL
17	BDL	BDL	5.5	4.5	BDL
18	BDL	4.5	BDL	BDL	BDL
19	BDL	BDL	BDL	BDL	BDL
20	BDL	BDL	5.3	4.5	BDL
Mean ± SD	BDL	0.5 ± 1.6	4.3 ± 2.3	2.8 ± 2.2	BDL

*BDL* below detection limit. BDL values on days 1 (+ 1), 4 (+ 2), 8 (+ 2) were considered as “0” in the calculation of the mean ± SD values

^a^Subject 12 discontinued the study on day 20. This subject completed study treatment (days 1–7), whereas assessments at days 14(+ 2), 17(+ 2) and 20(+ 2) were not performed

^b^Day 11 (+ 2) only

In general, most of the viable LCDG cells were isolated during the week of probiotic treatment, with a maximum count (mean log_10_ CFU/g of 4.3 ± 2.3 [range 3.7–6.3]; Table 3) reached between day 4 and 6 after the beginning of the intake.

For 3 of the 19 children with viable cells (15.8%), LCDG was already detected on day 3 (assessment time: day 1 [+ 2]) at counts of 4–4.8 log_10_ CFU/g, whereas for the other 17 children no viable LCDG was detectable at this time point.

Notably, for 11 (57.9%) of the 19 children with detectable live cells, LCDG survived in faecal samples up to 3 days after treatment end (day 10, i.e. assessment time: day 8 [+ 2]; Tables 2, 3). At this time-point, viable LCDG counts ranged from 3.7 to 5.5 log_10_ CFU/g, with a mean log_10_ of 2.8 ± 2.2 CFU/g.

### Defecation frequency and stool consistency

**Fig. 2:**
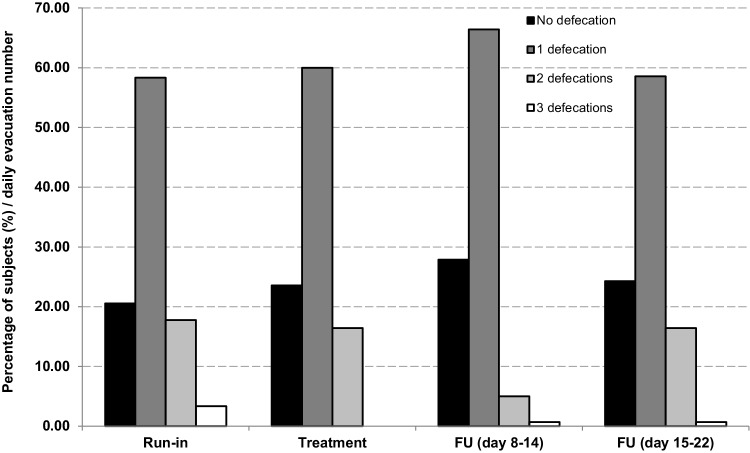
Average percentage of children reporting 0, 1, 2 or 3 defecations/day during the run-in, treatment and follow-up (days 8–14 and 15–22) study phases. *N* = 20

Weekly average daily defecation numbers are consistent throughout the study periods (Fig. 2). Percentage of subjects reporting 0, 1, 2 or 3 evacuations during the day did not change considerably from the run-in to the administration period and from the administration period to the follow-up, with most subjects reporting one defecation/day throughout the study.

**Fig. 3:**
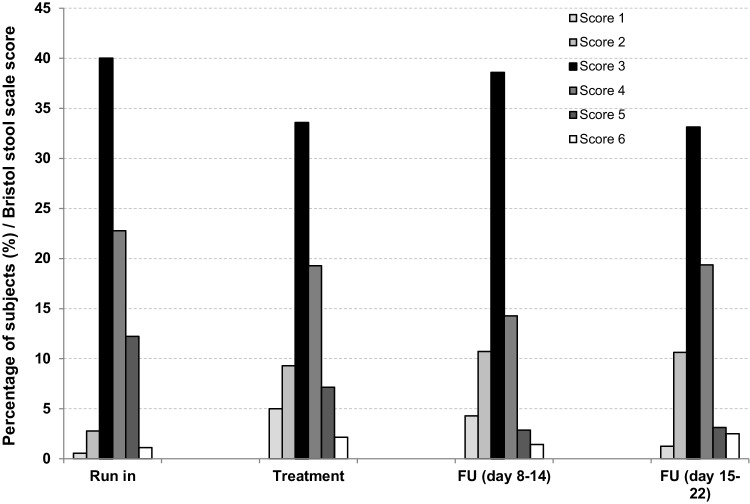
Average children percentage data for each stool consistency score, assessed daily using the Bristol 1–6 score scale*, during the run-in, treatment and follow-up (days 8–14 and 15–22) study phases. *N* = 20. *Score 1: separate hard lumps like nuts; score 2: sausage-shaped but lumpy; score 3: like a sausage but with cracks on the surface; score 4: like a sausage or snake, smooth and soft; score 5: soft blobs with clear-cut edges; score 6: fluffy pieces with ragged edges, a mushy stool; score 7: watery, no solid pieces, entirely liquid

The most frequent stool consistency score was 3 during most study days (Fig. 3). Scores 1 and 6 were seldom recorded (frequency < 5%) and score 7 was never recorded. Score 2 slightly increased and score 5 slightly decreased with time, during and after treatment.

Consistent with the overall evidence on defecation frequency and stool consistency, the children scored their digestive function most frequently as “Optimal” both at baseline (50%) and at the end of the administration period (55%), with the majority of the children who had an “Optimal” digestion at baseline maintaining the same digestive function during all study periods. Digestion was “Good” for 30% of the children at baseline and for 25% at study end. Notably, for one child who had a “Good” digestion at baseline digestion improved to “Optimal” starting from day 4 up to the last assessment (day 8). For the remaining children, digestive function was graded as “Normal”, with one child improving from “Normal” at baseline to “Good” at study end. No children scored their digestive function as “Bad” at any evaluation.

### Global evaluation and safety assessments

The individual global evaluation of the product intake was very good for 15 out of the 20 (75%) children. Of the other children, 3 (15%) judged product intake as good and 2 (10%) as normal.

The investigational product, administered to the study children once daily for 7 days, showed a very good safety profile. Only 4 subjects (20%) reported mild to moderate treatment-emergent adverse events either at the end of the treatment period or during the follow-up phase. The most common event was headache experienced by 2 (10%) children. All other adverse events (i.e. oropharyngeal pain, abdominal discomfort, pyrexia chills and tooth abscess) were reported by 1 (5%) subject each. The reported adverse events were judged as not related to study product intake, the majority of them were flu symptoms, and all resolved before study end. No clinically relevant findings were observed at the physical examination performed at the final visit.

## Discussion

In the present study, we have demonstrated for the first time that LCDG is capable of surviving the transit through the gastrointestinal tract of 3–12-year-old children during and after a 1-week consumption of a drinkable paediatric formulation, administered at the daily dose of 1 × 10^9^ CFU.

Nineteen (19) of the 20 treated children (95%) had LCDG CFU in their faecal samples during the administration period, 3 of them already after 1–3 days of treatment. Maximum viable LCDG counts were found at day 4–6 (mean 4.3 log_10_ CFU/g ± 2.3 [range 3.7–6.3 log_10_ CFU/g]).

These results confirm the ability of LCDG strain to pass the gastrointestinal barrier, i.e. to survive the untoward actions of gastric acid, bile acids and hydrolytic enzymes, also in children. According to these findings, in vitro results have previously shown that LCDG can resist at extreme pH (as low as pH 3) and bile acids conditions [19, 23].

Although no previous studies evaluated the survival of LCDG in children, a few studies were performed in infants who were administered other lactobacilli strains with different formulations. In a study performed in 2 months–6 years old children suffering from acute diarrhoea and administered for 5 days *L. rhamnosus* 573L/1, 573L/2, 573L/3 strains as milk/glucose solution (1.2 × 10 CFU; strain 1:1:1 proportion), viable bacterial cells were detected on the last treatment day in faeces samples of 37 out of the 46 (80.4%) treated children [33].

In another study, Marzotto et al. [34] observed that 92% of 26 (12–24-month-old) infants retained viable *L. paracasei* A cells, at counts ranging from 4.3 to 8.2 log_10_ CFU/g after the first week of consumption of 100 g fermented milk containing 8.2 log_10_ CFU/g of this *Lactobacillus* strain. As also previously reported, in fact, in most cases, ingested strains are still detected after a few days [35, 36]. In the above cited study [34], the percentage of children with positive samples decreased to 16% during the wash-out that followed the overall 4-week treatment. Notably, in the present study, live LCDG in faeces was present up to day 10, i.e. 3 days following the last product intake, in 57.9% of the study children at counts ranging from 3.7 to 5.5 log_10_ CFU/g, indicating a rather sustained persistence.

For comparison, in a study conducted in healthy adult volunteers [20] continuing their usual diet throughout the investigation, administration of a probiotic capsule containing at least 24 × 10^9^ viable LCDG, every day for 4 weeks, resulted in a significant increase (*p* < 0.001) in bacterial cells, detected in faecal samples of all subjects at the end of the probiotic intervention at a mean count of 7.5 ± 0.7 log_10_ CFU/g (range 6.2–8.3 log_10_ CFU/g), as compared to baseline (7/12 subjects; mean 5.1 ± 0.3 log_10_ CFU/g; range 4.7–5.6 log_10_ CFU/g). Interestingly, after a 4-week washout period, the LCDG cell number decreased to the amount before probiotic intake. More recently, the ability of LCDG to survive gastrointestinal transit in healthy adults after 1-week consumption of 1 × 10^9^ CFU per dose was evaluated [30]. The main finding of the study was that all 20 enrolled subjects were positive at least once for LCDG alive cells in the faecal sample, with the highest concentration between 4 and 8 days from the beginning of probiotic consumption. Alive probiotic cells were countable up to 5 days after the end of the Enterolactis^®^ formulation intake.

In the study by Drago et al. [29], after administration of 8.5 × 10^9^ CFU LCDG to 12 healthy adult volunteers once daily for 7 days, viable cells were detected in all samples during consumption, with mean counts ranging from 1.2 × 10^5^ on day 3 to 2.3 × 10^6^ CFU/g on day 7, and 1 week after treatment cessation (mean 1.1 × 10^6^ CFU/g).

The results of the present study are also consistent with previously published data obtained with various lactobacilli strains where bacteria were found in numbers ranging from < 2 log_10_ CFU/g to 8 log_10_ CFU/g [see e.g. 15–19, 29, 34, 36, 37].

In this study, 19 of the 20 enrolled and treated children were positive for viable LCDG cells at least once. Children 3 and 5 were found positive only during the follow-up phase, likely because recovery of bacteria in faecal samples is consistently variable between individuals [4]. Unexpectedly, for subject 10 a higher number of viable LCDG cells in faeces were found during the follow-up rather than during the week of treatment. As in the other referenced studies, a high variability in recovered live cells in faecal samples was observed. It is known that the diet can indirectly affect the survival of ingested probiotics [38]. The different amount of recovered LCDG cells in different subjects may thus be associated with the food consumed, which could affect the gastric emptying rate, and thus the survival of the probiotics [39], although other factors could have contributed to the variability observed. Faecal presence of ingested strains, also referred to as persistence, reflects not only the dose of the ingested strain, but also the extent of cell death (mainly in the upper gastrointestinal tract), and the subsequent replication of surviving cells.

In the present study, digestive function was also evaluated, to assess whether LCDG intake for a short time period and in a healthy paediatric population could already exert a beneficial effect. Results showed that digestive function was reported as “Optimal” or “Good” for the majority of subjects already before the consumption of the investigational product. The digestive function either did not change (for 18/20 children) or improved only very slightly and only for 2 children at the end of the 1-week administration period as compared to baseline.

In addition, the majority of subjects reported one stool evacuation each day during the whole study duration, with negligible changes in defecation frequency between the study periods. Stool consistency did not significantly change during the study, with score 3 (*like a sausage but with cracks on the surface*) being the most frequent at all assessment times. To note that score 3 is an indicator of a satisfactory stool consistency. Upon treatment, score 2 (*sausage-shaped but lumpy*) slightly increased and score 5 (*soft blobs*) slightly decreased, suggesting a very modest digestion improvement, although not clinically relevant, during and after treatment. Based on currently available evidence, *L. rhamnosus* GG strain has proven to be efficacious in the treatment of children acute gastroenteritis, prevention of antibiotic-associated diarrhoea and prevention of nosocomial diarrhoea [27, 40–42]. In addition, similar to the findings of the present investigation, a previous study in healthy adults showed that a 2-week administration of fermented milk containing a strain of *L. casei* (i.e. *L. casei* Shirota) did not change bowel movements frequency or stool consistency [18].

In the present study, general digestive conditions of the enrolled healthy children, including defecation frequency, stool consistency and digestive function, were already satisfactory at study entry, due to the restrictions imposed by the study inclusion criteria. It is likely that this, together with the short administration period, could be the reason why no relevant changes were observed upon probiotic treatment.

In the present study, the good safety profile and palatability of LCDG drinkable paediatric formulation were also confirmed.

In conclusion, the present preliminary study, carried out in healthy children, aged 3–12 years, demonstrated for the first time that L. casei DG^®^ survives the gastrointestinal transit when ingested with the paediatric probiotic drinkable formulation containing 1 × 10^9^ CFU, and persists in the gut up to 3 days after the end of probiotic consumption, demonstrating resistance to gastric juices, hydrolytic enzymes and bile acids.

## Electronic supplementary material

Below is the link to the electronic supplementary material.

Supplementary material 1 (DOCX 18 KB)

## Abbreviations

CFU: Colony forming unit
GCP: Good clinical practice
GI: Gastrointestinal
GRAS: Generally recognized as safe
ICH: International Conference on Harmonisation
LCDG: L. casei DG^®^
