# Supplementary material for: Survival of L. casei DG® (Lactobacillus paracasei CNCMI1572) in the gastrointestinal tract of a healthy paediatric population
Source: Eur J Nutr. 2018 Nov 29;58(8):3161–70. doi: 10.1007/s00394-018-1860-5 (PMC6842349; doi:10.1007/s00394-018-1860-5)
Supplement: Supplementary file 1 — Supplementary material 1 (DOCX 18 KB) [file 394_2018_1860_MOESM1_ESM.docx]

Supplementary Table S1 Individual demography parameters and measured data up to day 8 (+2)

| **Subject** | **Demography** | | **BMI (kg/m^2^)** | **Viable L. casei DG^®^ counts (log10 CFU/ g faeces)** | | | |
| --- | --- | --- | --- | --- | --- | --- | --- |
|  |  |  |  | **Baseline** | **One-week administration period** | | **Follow-up** |
|  | **Sex (10F/10M)** | **Age (3-12 years)** |  | **Day -2 (+1)** | **Day 1 (+2)** | **Day 4 (+2)** | **Day 8 (+2)** |
| 1 | F | 9 | 18.4 | BDL | BDL | 5.7 | 3.7 |
| 2 | M | 10 | 17.8 | BDL | BDL | 4.5 | BDL |
| 3 | F | 3 | 16.5 | BDL | BDL | BDL | 5.5 |
| 4 | F | 9 | 15.4 | BDL | BDL | 5.7 | BDL |
| 5 | F | 4 | 15.6 | BDL | BDL | BDL | 4.7 |
| 6 | F | 9 | 15.8 | BDL | BDL | 4.7 | BDL |
| 7 | M | 12 | 20.6 | BDL | BDL | 5.9 | BDL |
| 8 | F | 9 | 18.3 | BDL | BDL | 5.3 | 4.7 |
| 9 | F | 3 | 15.2 | BDL | 4 | 6.3 | 4 |
| 10 | M | 6 | 17.2 | BDL | BDL | 3.7 | 4.7 |
| 11 | F | 12 | 20.9 | BDL | BDL | 5 | 3.95 |
| 12* | M | 4 | 14.2 | BDL | BDL | 5.3 | BDL |
| 13 | F | 7 | 14.6 | BDL | BDL | 5.9 | 3.3 |
| 14 | F | 5 | 14.3 | BDL | BDL | 5 | 4.7 |
| 15 | M | 8 | 16.2 | BDL | 4.8 | 5.3 | 4 |
| 16 | M | 6 | 15.7 | BDL | BDL | 5.9 | 4.3 |
| 17 | M | 5 | 15.4 | BDL | BDL | 5.5 | 4.5 |
| 18 | M | 6 | 15.4 | BDL | 4.5 | BDL | BDL |
| 19 | M | 4 | 15.3 | BDL | BDL | BDL | BDL |
| 20 | M | 9 | 16.9 | BDL | BDL | 5.3 | 4.5 |

** Subject 12 discontinued the study on day 20. This subject completed study treatment (days 1-7), whereas assessments at days 14(+2), 17(+2) and 20(+2) were not performed.*

*.*
